# Benzoic acid supplementation improves the growth performance, nutrient digestibility and nitrogen metabolism of weaned lambs

**DOI:** 10.3389/fvets.2024.1351394

**Published:** 2024-02-09

**Authors:** Wenjie Zhang, Shuo Sun, Yaqian Zhang, Yanan Zhang, Jianguo Wang, Zhiqiang Liu, Kailun Yang

**Affiliations:** ^1^Xinjiang Key Laboratory of Meat and Milk Production Herbivore Nutrition, College of Animal Science and Technology, Xinjiang Agricultural University, Ürümqi, China; ^2^Xinjiang Shangpin Meiyang Technology Co., Ltd., Changji, China

**Keywords:** benzoic acid, weaned lambs, hippuric acid, growth performance, nitrogen metabolism

## Abstract

Nitrogen is one of the essential components of proteins and nucleic acids and plays a crucial role in the growth and development of ruminants. However, the nitrogen utilization rate of ruminants is lower than that of monogastric animals, which not only reduces protein conversion and utilization, but also increases manure nitrogen discharge as well as causing environmental pollution. The lamb stage is an important period in the life of sheep, which can affect the production performance and meat quality of fattening sheep. The purpose of this experiment was to explore effects of benzoic acid supplementation on growth performance, nutrient digestibility, nitrogen metabolism and plasma parameters of weaned lambs. A total of 40 weaned male Hu sheep lambs with similar body weight were randomly divided into 4 groups: control with no benzoic acid (0 BA) and the lambs in other 3 groups were fed 0.5, 1, and 1.5% benzoic acid on the basis of experimental diet (0.5, 1, and 1.5 BA, respectively). The experiment lasted for 60 days. Results showed that the average daily gain of 1 BA group was significantly increased (*p* < 0.05) when compared to 0 and 1.5 BA groups, while an opposite tendency of dry matter intake to average daily gain ratio was observed. The dry matter, organic matter, neutral detergent fiber and acid detergent fiber digestibility of 1 BA group was significantly increased (*p* < 0.05) as compared with 0 and 1.5 BA groups as well as plasma albumin content. Also, the urinary hippuric acid and hippurate nitrogen concentrations in 1 and 1.5 BA groups were higher (*p* < 0.05) than those in 0 and 0.5 BA groups. Additionally, the nitrogen intake in 0.5 and 1 BA groups was significantly increased (*p* < 0.05) when compared to other groups. At 1 h after morning feeding, the plasma benzoic acid concentration of 1 BA group reached up to maximum value and was higher (*p* < 0.05) than other groups, and then began to decrease. Similarly, the hippuric acid concentration in plasma of 1 and 1.5 BA groups was higher (*p* < 0.05) than that of 0 BA group from 1 to 4 h post morning feeding. At 3 h after feeding, the urea nitrogen concentration in plasma of 0 BA group was higher (*p* < 0.05) than that of 1.5 BA group. Overall, the appropriate supplementation of benzoic acid (1%) in the diet can improve growth performance and nitrogen metabolism of weaned lambs.

## 1 Introduction

With the shortage of feed resources and further aggravation of environmental pollution, it is difficult to increase animals' food production by simply expanding the number of breeding heads and ignoring environmental pollution. Technological innovation is one of the main ways to transform animals' production mode and improve animals' output rate and resource utilization rate, which are beneficial for establishment of resource-saving and environment-friendly society in the future (1). A large amount of nitrogen substances excreted by livestock is an important cause of environmental pollution in animal husbandry (2, 3). These nitrogen substances mainly derive from the undigested crude protein and degradation of amino acids in feed (4, 5). Nitrogen is one of the essential components of proteins and nucleic acids and plays a crucial role in the growth and development of ruminants. However, under the current dietary feeding system, the nitrogen utilization rate of ruminants is lower than that of monogastric animals (6). The nitrogen utilization rate of ruminants is approximately 20 to 36%, and the remaining 64 to 80% of nitrogen is excreted in the form of feces and urine (7), which not only reduces protein conversion and utilization, but also increases manure nitrogen discharge (8–10). Lower nitrogen utilization of ruminants severely restricts the economic benefits of livestock farms. Therefore, increasing the efficiency of nitrogen conversion and utilization in ruminants by nutritional strategy is of great significance for ruminants industry as well as reducing the environmental pollution (11).

Hippuric acid (HA), also known as benzoylglycine, is the glycine conjugate of benzoic acid (BA), and it is found in high concentrations in the urine of herbivores (12). For herbivores, including ruminants, polyphenols in plant-based diets can form BA under the joint action of gut microorganisms, which are subsequently absorbed through the gut and transported to the liver for metabolism (13). In animals' liver mitochondria, BA and glycine are catalyzed by enzymes to form HA, which is then excreted in the urine (14). In early research, Doak (15) found that urea nitrogen accounted for 76.4% of urinary nitrogen in wether, and the proportions of allantoin nitrogen and HA nitrogen were 4.1 and 2.6%, respectively. Bristow et al. study analyzed the nitrogen content in the urine of cattle and sheep, and found that 69% of the total nitrogen in bovine urine was in the form of urea, 7.3% in the form of allantoin, and 5.8% in the form of HA. In sheep urine, the nitrogen in the form of urea accounted for 83% of the total nitrogen content, and both HA nitrogen and allantoin nitrogen accounted for 4.3% of total nitrogen (16). Thus, nitrogen excreted by HA can be used as another way of nitrogen excretion in ruminants.

BA is an important precursor in the process of HA formation, and its content directly affects the excretion of HA. As a weakly acidic aromatic acid organic compound, BA is widely used as organic acidifier and preservative in food, medicine and feed industry because of its broad antibacterial effect and strong antibacterial ability (17, 18). BA can improve the growth performance and feed conversion of monogastric animals by promoting the production and activation of gastrointestinal digestive enzymes (19), enhancing gut absorption capacity (20), improving intestinal barrier (21) and regulating intestinal microbiota (22). Nevertheless, little attention has been paid to the effects of BA on urea metabolism, nitrogen metabolism and growth performance of ruminants. Noticeably, both HA synthesis and urea cycle occur in the liver mitochondria of ruminants. Among them, the urea cycle mainly consists of 1 molecule NH_3_ and 1 molecule CO_2_ catalyzed by carbamyl phosphate synthetase I to produce carbamyl phosphate (23). The NH${\;}_{4}^{+}$ produced by glutamine in mitochondria can be used to synthesize carbamyl phosphate as well as glycine. When the entry of BA into liver cells increases, HA synthesis is elevated, and the consumption of glycine correspondingly increases, thus up-regulating the synthesis of glycine in liver mitochondria. If the amount of NH${\;}_{4}^{+}$ in the mitochondria used to synthesize glycine increases, does the amount used to synthesize carbamyl phosphate decrease, then reducing urea production? However, there is a lack of relevant research at present. Therefore, this study was carried out to evaluate the effects of different levels of BA supplementation on nutrient digestibility, urea metabolism, nitrogen metabolism and growth performance of weaned lambs.

## 2 Materials and methods

### 2.1 Ethic statement

All animal care and handing procedures in this study were conducted under the guidance of the Care and Use of Laboratory Animals in China and were approved by (protocol number: 2020022) the Animal Care Committee of Xinjiang Agricultural University (Urumqi, Xinjiang, China).

### 2.2 Experimental animals and feeding management

The animal experiment was conducted at a commercial sheep farm located at Agricultural Science and Technology Park, Changji, China. A total of 40 healthy male Hu sheep lambs with similar age and body weight (BW, 17.27 ± 1.52 kg) after weaning were used. After marking with ear tags, the lambs were randomly allocated to 4 groups, each with 10 animals. All lambs were fed a same basal diet that was formulated according to the NRC (24). Feed compositions and nutrient levels of experimental diet are presented in Table 1. Lambs in each group were supplemented with 0, 0.5, 1 and 1.5% BA (Purchased from Henan Xizheng Industry Co., China; Purity ≥ 99.5%) in the basal diet, and the treatments were labeled as 0, 0.5, 1, and 1.5 BA groups, respectively. The additive amount of BA was based on the previous studies in beef cattle (25) and grow-finisher pigs (26).

**Table 1:** Feed ingredients and nutrient levels of the diet (DM basis).

**Ingredients, %**	**Content**	**Nutrient levels^b^, %**	**Content**
Corn	11.88	DM	93.04
Wheat bran	4.15	OM	95.61
Soybean meal	6.47	CP	15.03
Stone powder	0.23	EE	4.14
NaCl	0.12	NDF	26.40
Premix^a^	0.23	ADF	12.44
Alfalfa hay	38.46	Ca	1.02
Whole corn silage	38.46	P	0.36
Total	100.00		

DM, dry matter; OM, organic matter; CP, crude protein; EE, ether extract; NDF, neutral detergent fiber; ADF, acid detergent fiber; ^a^The Premix provided the following per kg of diets: S (sulphur) 200 mg, Fe (ferrous) 25 mg, Zn (zinc) 40 mg, Cu (cuprum) 8 mg, Mn (manganese) 40 mg, I (iodine) 0.3 mg, Se (selenium) 0.2 mg, Co (cobalt) 0.1 mg, VA 940 IU, VD 111 IU, VE 20 IU; ^b^Nutrient levels were measured values.

The current study was performed from February to April of 2023. All animals of 4 treatments were reared in 40 pens with 1 lamb in each pen (1 × 1.2 m). The 40 pens were located inside a barn open on two sides and arranged in two rows of 20, separated by the central feeding lane. The pens are enclosed by horizontal metal rail bars, which also delimit the pens at the feeding lane. The floor had a concrete base covered with barley straw bedding, of which one fresh flake (around 1.5 kg) per pen was added over the permanent bedding once a day. The lambs were untethered and did not have any access to a paddock area. BA was fully mixed with the basal diet. Lambs were fed twice daily at 10:00 and 17:00, respectively, allowing 5 to 10% orts, and given free access to drinking clean water. Before feeding trail, the experimental shed was cleaned and sterilized, and parasites were eliminated. A 5-d adaptive phase was followed by 60 days of experimental period.

### 2.3 Growth performance measurement

Before morning feeding, the BW of all lambs was measured on d 0, 30, and 60, and the average daily gain (ADG) was calculated by initial and final BW. The feed intake was recorded according to the difference of feed offered and refused and converted into dry matter intake (DMI). Feed conversion ratio (F:G) was determined through dividing DMI by ADG.

### 2.4 Urine, feces and blood samples collection

From d 50 to 55 of the experiment, 6 lambs in each group were randomly selected to collect urine and fecal samples. A self-made urine collection device was used to collect lamb urine samples, and urine was collected every 4 h throughout the day to record the daily urine output of lambs in detail. All the urine of per lamb during the digestion and metabolism experiment were fully shook. The total urine weight was determined by an electronic balance (Deante Sensor Technology Co., Ltd., Tianjin, China), and 10% of the total urine was subsampled and stored in urine sample bottles. Immediately, the pH of urine was determined by a portable pH meter (Ruibin Technology Co., Ltd., Guangzhou, China). Next, the per 100 mL of urine samples were mixed with 10 mL of 10% sulphuric acid for acidification (27) and preserved at−20°C for analysis of BA content and nitrogen metabolism.

In addition, fecal samples were collected in nylon sieve plates placed under the floor of the individual lamb stall. The feces were collected every 4 h throughout the day. After the lamb fecal samples were thoroughly mixed for 6 consecutive days, 10% of the total amount was randomly weighed. Meanwhile, the fresh feed and orts were sampled daily. The fecal samples were mixed per lamb and subsampled. All feed, orts and fecal samples (the 100 g feces were mixed with 10 mL of 10% sulphuric acid) were dried at 65°C in a forced-air oven (Hengmai drying equipment Co., Ltd., Changzhou, China) for 48 h to a constant weight. Then, air-dried samples were ground to pass through a 1-mm sieve (Xulang machinery Equipment Co., Ltd., Guangzhou, China) for measurement of nitrogen metabolism and apparent digestibility.

Before morning feeding (0 h) and 1, 2, 3, and 4 h after morning feeding on d 40, 6 lambs from each treatment were randomly selected to collect blood samples. During each sampling time point, a total of 5 mL blood was sampled from the jugular vein of each lamb using evacuated tubes containing no anticoagulant. Then, blood samples were centrifuged at 3,500 × *g* and 4°C for 15 min to collect plasma. The plasma was stored at−20°C for further analysis.

### 2.5 Urine, feces and blood samples analysis

The feed and fecal samples were analyzed for DM (method 934.01), organic matter (OM, method 942.05), CP (method 990.93), ether extract (EE, method 920.39), Calcium (Ca) and Phosphorus (P) reference to the AOAC procedures (28). In addition, the neutral detergent fiber (NDF) and acid detergent fiber (ADF) contents were determined using an ANKOM fiber analyzer (A2000i, Ankom Technology Corp., Macedon, New York, USA). The chemical composition contents in feed and feces, and DMI and fecal weight were used to calculate the apparent digestibility (29).

Plasma samples collected on d 40 before morning were used to measure contents of biochemical parameters, including glucose (GLU), total bilirubin (T-Bil), direct bilirubin (D-Bil), alanine transaminase (ALT), aspartate transaminase (AST), alkaline phosphatase (ALP), glutamyl transferase (GT), total protein (TP), triglyceride (TG), total cholesterol (TC) and albumin (ALB), with an automatic biochemical analyzer (ZY KHB-1280, Huaren Biotechnology Co., Ltd., Nanjing, China). Plasma samples collected at dynamic points were used for the determination of BA, HA and urea nitrogen. The BA and HA concentrations in plasma and urine were analyzed by high-performance liquid chromatography (HPLC) following the procedures of Kubota et al. (30). Briefly, 100 μL of plasma (200 μL of urine after 10-fold dilution) was transferred to another centrifuge tube, and 200 μL (400 μL of urine) acetonitrile precipitated protein containing o-chlorobenzoic acid as the internal target was added. The samples were swirled and mixed for 20 s, and centrifuged at 9,500 × *g* for 1 min. Subsequently, 10 μL of mixture was collected and measured using liquid chromatograph. Determination conditions: IC YS-50 weakly acidic cation exchange column 4.6 × 125 mm was used; flow rate 1.0 mL/min; column temperature 30°C; detection wavelength 235 nm; a linear elution; sample size was 10 μL. In addition, urea nitrogen was determined using commercial kit (NO.RATA-A 7170 Huaying, Beijing, China) reference to the instructions.

### 2.6 Statistical analysis

All data were analyzed with one-way ANOVA procedure of the SPSS statistical software (version 22.0 for Windows; SPSS, Chicago, USA), with each lamb as an experimental unit. Orthogonal polynomial contrasts were completed to detect the linear and quadratic effects of benzoic acid levels. Duncan test was conducted to determine the differences among four treatments. Data were presented as mean and standard error of mean. The significance level was indicated at *p* ≤ 0.05, and a trend was declared at 0.05 < *p* ≤ 0.10. Besides, the dynamic changes of BA, HA and urea nitrogen were used to draw broken line graphs using GraphPad Prism software (version 8.0 for Windows; GraphPad Prism, San Diego, USA).

## 3 Results

### 3.1 Growth performance of weaned lambs

As shown in Table 2, the initial BW and BW on d 30 did not show significant difference (*p* > 0.05) among all groups. However, the final BW showed quadratic variation tendency (*p* = 0.006), and the 1 BA group had the highest value. The DMI and ADG were similar (*p* > 0.05) among all groups from d 1 to 30, and F:G was significantly lower in the 1BA group as compared to the 0 BA group (*p* < 0.05). From d 31 to 60, the DMI and ADG of 1 BA group were higher (*p* < 0.05) than those of 0 BA and 1.5 BA groups, whereas F:G displayed an opposite trend. No significant difference (*p* > 0.05) of DMI was observed among all group during the whole experimental period. Compared with 0 BA and 1.5 BA groups, the ADG in 1 BA group was significantly elevated (*p* < 0.05) from d 1 to 60. The F:G of 1 BA group had minimum value and lower (*p* < 0.05) than 0 and 1.5 BA groups.

**Table 2:** Effects of benzoic acid supplementation on dry matter intake and average daily gain of weaned lambs.

**Items**	**Groups**				**SEM**	***P-*** **value**		
	**0 BA**	**0.5 BA**	**1 BA**	**1.5 BA**		**Treatment**	**Linear**	**Quadratic**
**Body weight, kg**								
Initial BW	17.24	17.26	17.33	17.27	0.24	0.999	0.947	0.934
d 30 BW	21.05	21.85	22.70	21.01	0.41	0.428	0.840	0.138
Final BW	26.84^b^	29.32^ab^	30.93^a^	27.02^b^	0.59	0.034	0.662	0.006
**Day 1 to 30**								
DMI, g/d	601.80	599.29	598.35	579.71	10.99	0.890	0.500	0.717
ADG, g/d	127.10	153.00	179.17	125.00	9.87	0.172	0.818	0.044
F:G	4.73^a^	3.92^ab^	3.34^b^	4.64^a^	0.13	0.083	0.672	0.004
**Day 31 to 60**								
DMI, g/d	834.35^b^	861.27^ab^	873.85^a^	828.12^b^	6.64	0.043	0.917	0.006
ADG, g/d	192.83^b^	248.83^a^	274.33^a^	200.00^b^	9.55	0.003	0.525	< 0.001
F:G	4.33^a^	3.46^ab^	3.19^b^	4.14^a^	0.15	0.031	0.342	0.002
**Day 1 to 60**								
DMI, g/d	718.07	730.28	736.10	703.02	10.42	0.710	0.656	0.320
ADG, g/d	159.97^b^	200.92^ab^	226.75^a^	162.50^b^	8.52	0.009	0.624	0.001
F:G	4.49^a^	3.63^ab^	3.25^b^	4.33^a^	0.12	0.055	0.024	0.008

BA, benzoic acid; BW, body weight; DMI, dry matter intake; ADG, average daily gain; SEM, standard error of mean. 0 BA, control with no BA (Henan, China); 0.5 BA, supplementation of 0.5% BA on the basis of experimental diet; 1 BA, supplementation of 1% BA on the basis of experimental diet; 1.5 BA, supplementation of 1.5% BA on the basis of experimental diet. In the same row, values with different superscripts differ significantly (p < 0.05). F:G, DMI/ADG.

### 3.2 Nutrients digestibility of weaned lambs

Obviously, the apparent digestibility of CP, EE, Ca and P was similar (*p* > 0.05) among four groups. The DM, OM and ADF digestibility of 1 BA group were higher (*p* < 0.05) than those of 0 BA group (Table 3). Compared with other groups, the NDF digestibility in 1 BA group was significantly increased (*p* < 0.05).

**Table 3:** Effects of benzoic acid supplementation on nutrients digestibility of weaned lambs.

**Items**	**Groups**				**SEM**	***P*** **-value**		
	**0 BA**	**0.5 BA**	**1 BA**	**1.5 BA**		**Treatment**	**Linear**	**Quadratic**
DM	64.19^b^	67.41^a^	70.12^a^	65.83^ab^	0.71	0.011	0.143	0.004
OM	68.55^c^	72.12^ab^	73.84^a^	70.37^bc^	0.64	0.009	0.120	0.002
CP	59.09	61.28	65.42	64.03	1.10	0.174	0.057	0.398
NDF	64.77^b^	65.56^b^	69.55^a^	64.49^b^	0.73	0.033	0.568	0.029
ADF	60.15^c^	64.69^ab^	66.49^a^	62.79^bc^	0.68	0.001	0.029	< 0.001
EE	73.99	79.49	81.03	75.03	1.11	0.052	0.592	0.008
Ca	44.80	46.59	51.99	47.36	1.25	0.215	0.236	0.196
P	39.16	43.86	47.83	41.29	1.57	0.249	0.455	0.082

BA, benzoic acid; DM, dry matter; OM, organic matter; CP, crude protein; NDF, neutral detergent fiber; ADF, acid detergent fiber; EE, ether extract; SEM, standard error of mean. 0 BA, control with no BA (Henan, China); 0.5 BA, supplementation of 0.5% BA on the basis of experimental diet; 1 BA, supplementation of 1% BA on the basis of experimental diet; 1.5 BA, supplementation of 1.5% BA on the basis of experimental diet. In the same row, values with different superscripts differ significantly (p < 0.05).

### 3.3 Urinary pH and nitrogen excretion of weaned lambs

No obvious difference (*p* > 0.05) of urinary pH, output, total nitrogen excretion and other ingredients nitrogen excretion was found among four groups (Table 4). The HA and hippurate nitrogen in 1 and 1.5 BA groups were higher (*p* < 0.05) than those in 0 and 0.5 BA groups. Likewise, compared with 0 BA group, the HA excretion and hippurate nitrogen excretion in BA treatments were significantly increased (*p* < 0.05). An opposite trend of urea nitrogen was observed between 0 BA group and BA treatments. However, the urea nitrogen excretion was similar (*p* > 0.05) among all groups. In addition, total nitrogen and other ingredients nitrogen in urine of 1.5 BA group were higher (*p* < 0.05) than that of 0 and 0.5 BA groups.

**Table 4:** Effects of supplementing different levels of benzoic acid on urinary pH and nitrogen excretion in weaned lambs.

**Items**	**Groups**				**SEM**	***P*** **-value**		
	**0 BA**	**0.5 BA**	**1 BA**	**1.5 BA**		**Treatment**	**Linear**	**Quadratic**
Urinary pH	6.65	6.58	6.54	6.52	0.02	0.174	0.003	0.346
Urinary output, mL	460.83	487.27	480.40	451.77	39.71	0.138	0.008	0.105
Hippuric acid, mg/mL	6.59^c^	20.95^b^	27.61^a^	28.20^a^	1.86	< 0.001	< 0.001	< 0.001
Hippuric acid excretion, g/d	2.84^b^	9.89^a^	13.29^a^	12.60^a^	1.26	0.005	0.001	0.064
Hippurate nitrogen, g/L	0.51^c^	1.64^b^	2.16^a^	2.20^a^	0.15	< 0.001	< 0.001	< 0.001
Hippurate nitrogen excretion, g/d	0.22^b^	0.77^a^	1.04^a^	0.98^a^	0.10	0.005	0.001	0.064
Urea nitrogen, g/L	5.73^a^	4.87^b^	4.75^b^	4.68^b^	0.23	0.041	0.013	0.155
Urea nitrogen excretion, g/d	2.68	2.44	2.28	1.99	0.15	0.741	0.278	0.959
Other ingredients nitrogen, g/L	1.63^b^	1.56^b^	3.12^ab^	4.55^a^	0.46	0.057	0.011	0.374
Other ingredients nitrogen excretion, g/d	0.66	0.72	1.42	1.59	0.17	0.108	0.022	0.856
Total nitrogen in urine, g/L	7.88^b^	8.07^b^	10.00^ab^	11.44^a^	0.54	0.048	0.008	0.521
Urinary total nitrogen excretion, g/d	3.56	3.93	4.74	4.57	0.37	0.671	0.271	0.728

BA, benzoic acid; SEM, standard error of mean. 0 BA, control with no BA (Henan, China); 0.5 BA, supplementation of 0.5% BA on the basis of experimental diet; 1 BA, supplementation of 1% BA on the basis of experimental diet; 1.5 BA, supplementation of 1.5% BA on the basis of experimental diet. In the same row, values with different superscripts differ significantly (p < 0.05).

### 3.4 Correlation analysis of hippurate nitrogen and urea nitrogen in weaned lambs

**Figure 1:**
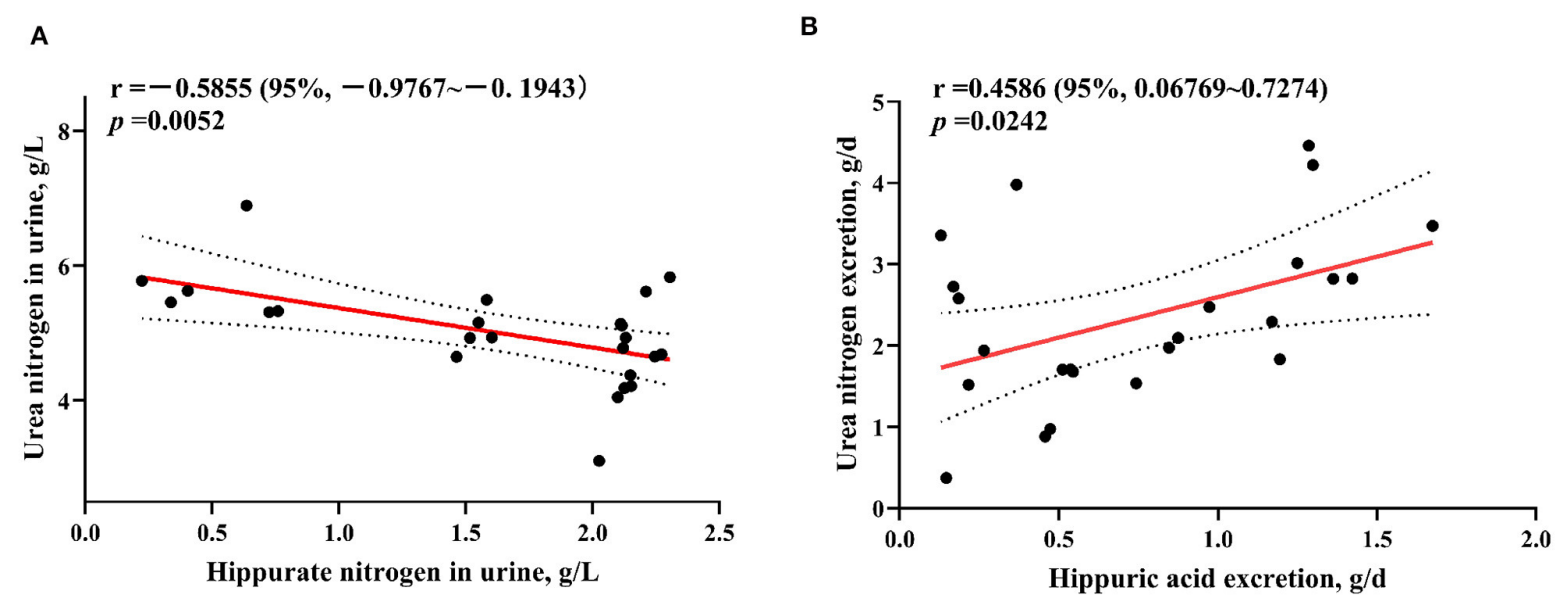
Analysis of the content of hippurate nitrogen and urea nitrogen in the urine of weaned lambs **(A)** and the correlation between hippurate nitrogen and urea nitrogen excretion **(B)**.

As can be seen in Figure 1, the HA nitrogen content in the urine of lambs was significantly negatively correlated with the urea nitrogen content (*p* = 0.0052), and the amount of HA nitrogen excreted was significantly positively correlated with the amount of urea nitrogen excreted (*p* = 0.0242).

### 3.5 Nitrogen metabolism of weaned lambs

There was no significant difference (*p* > 0.05) of fecal nitrogen, urinary nitrogen and nitrogen retention among all groups (Table 5). Nevertheless, the nitrogen intake in 0.5 and 1 BA groups was significantly enhanced (*p* < 0.05) when compared to 0 and 1.5 BA groups.

**Table 5:** Effects of supplementing different levels of benzoic acid on nitrogen metabolism in weaned lambs (g/d).

**Items**	**Groups**				**SEM**	***P*** **-value**		
	**0 BA**	**0.5 BA**	**1 BA**	**1.5 BA**		**Treatment**	**Linear**	**Quadratic**
Nitrogen intake	18.75^b^	19.80^a^	20.35^a^	18.69^b^	0.20	0.001	0.746	< 0.001
Fecal nitrogen	7.63	7.65	7.04	6.72	0.18	0.187	0.054	0.626
Urinary nitrogen	3.56	3.93	4.74	4.57	0.37	0.671	0.271	0.728
Nitrogen retention	7.56	8.21	8.57	7.41	0.40	0.257	0.626	0.118

BA, benzoic acid; SEM, standard error of mean. 0 BA, control with no BA (Henan, China); 0.5 BA, supplementation of 0.5% BA on the basis of experimental diet; 1 BA, supplementation of 1% BA on the basis of experimental diet; 1.5 BA, supplementation of 1.5% BA on the basis of experimental diet. In the same row, values with different superscripts differ significantly (p < 0.05).

### 3.6 Blood biochemical parameters of weaned lambs

The concentrations of GLU, T-Bil, D-Bil, ALT, AST, ALP, GT, TP, TG and TC in plasma were similar (*p* > 0.05) among four groups (Table 6). Compared with 0 BA and 1.5 BA groups, the plasma ALB content of 1 BA group was significantly increased (*p* < 0.05).

**Table 6:** Effects of supplementing different levels of benzoic acid on blood biochemical parameters in weaned lambs.

**Items**	**Groups**				**SEM**	***P*** **-value**		
	**0 BA**	**0.5 BA**	**1 BA**	**1.5 BA**		**Treatment**	**Linear**	**Quadratic**
GLU, mmol/L	5.17	4.90	5.40	5.29	0.12	0.536	0.451	0.768
T-Bil, μmol/L	3.46	3.26	3.30	3.18	0.09	0.725	0.329	0.808
D-Bil, μmol/L	1.15	1.18	1.16	1.16	0.03	0.990	0.964	0.850
ALT, U/L	15.53	13.77	15.87	13.97	0.91	0.817	0.765	0.973
AST, U/L	87.50	105.97	98.22	104.22	3.87	0.339	0.229	0.424
ALP, U/L	326.45	236.85	293.27	214.87	21.90	0.260	0.159	0.897
GT, U/L	71.83	69.13	75.22	66.75	2.44	0.673	0.689	0.574
TP, g/L	49.12	52.18	49.95	54.58	0.94	0.159	0.089	0.663
TG, mmol/L	0.36	0.34	0.37	0.37	0.02	0.956	0.845	0.750
TC, mmol/L	1.21	1.22	1.36	1.28	0.03	0.295	0.199	0.472
ALB, g/L	23.23^b^	24.22^ab^	25.68^a^	22.87^b^	0.40	0.044	0.614	0.204

BA, benzoic acid; GLU, glucose; T-Bil, total bilirubin; D-Bil, direct bilirubin; ALT, alanine transaminase; AST, aspartate transaminase; ALP, alkaline phosphatase; GT, glutamyl transferase; TP, total protein; TG, triglyceride; TC, total cholesterol; ALB, albumin; SEM, standard error of mean. 0 BA, control with no BA (Henan, China); 0.5 BA, supplementation of 0.5% BA on the basis of experimental diet; 1 BA, supplementation of 1% BA on the basis of experimental diet; 1.5 BA, supplementation of 1.5% BA on the basis of experimental diet. In the same row, values with different superscripts differ significantly (p < 0.05).

### 3.7 Dynamic changes of benzoic acid, hippuric acid and urea nitrogen in plasma of weaned lambs

**Figure 2:**
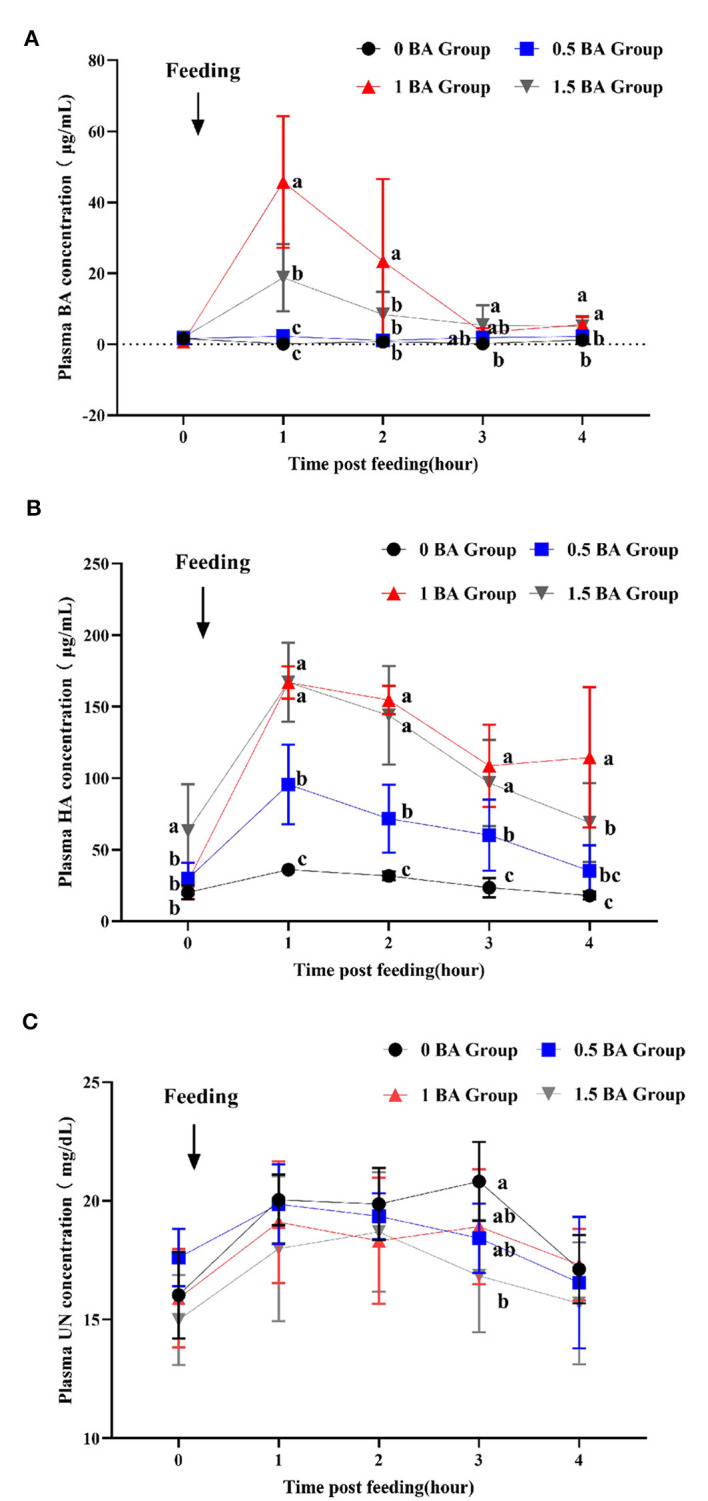
Effects of supplementing different levels of benzoic acid on dynamic changes of benzoic acid **(A)**, hippuric acid **(B)**, and urea nitrogen **(C)** in plasma in weaned lambs. BA, benzoic acid; HA, hippuric acid; UN, urea nitrogen. 0 BA, control with no BA (Henan, China); 0.5 BA, supplementation of 0.5% BA on the basis of experimental diet; 1 BA, supplementation of 1% BA on the basis of experimental diet; 1.5 BA, supplementation of 1.5% BA on the basis of experimental diet. Different letters (a, b, and c) represent statistically significant differences (*P* < 0.05).

As shown in Figure 2A, at 1 h after morning feeding, the plasma BA concentration of 1 BA group reached up to maximum value and was higher (*p* < 0.05) than other groups, and then began to decrease. At 1 post feeding, the concentration of BA in 1.5 BA group was significantly increased (*p* < 0.05) as compared with 0 and 0.5 BA groups. During the whole sampling process, the BA content in 0 and 0.5 BA groups was always at a lower level. Similarly, the HA concentration in plasma of 1 and 1.5 BA groups was higher (*p* < 0.05) than that of 0 BA group from 1 to 4 h post morning feeding (Figure 2B). After lambs supplemented with BA, the plasma HA concentration gradually increased and reached the highest value at 1 h post feeding and then began to display a fluctuation change. The HA concentration of 0, 0.5, and 1.5 BA groups at 4 h was close to corresponding 0 h. On the contrary, at 3 h after feeding, the urea nitrogen concentration in plasma of 0 BA group was higher (*p* < 0.05) than that of 1.5 BA group (Figure 2C). No obvious difference (*p* > 0.05) of urea nitrogen concentration was observed at other time points among all groups.

## 4 Discussion

Lamb nutrition is an increasingly important issue in today's sheep production. The feeding management of lambs has long-term influence on future production performance of meat quality of fattening sheep (31). Due to the immature gastrointestinal tracts, the lambs are easily affected by harmful microorganisms. After weaning, lambs undergo the changes of feed type and rearing pattern, resulting in decreased nutrient digestibility and growth rate (32). Relieving the weaning stress of lambs is of great significance for improving the healthy growth of lambs. As an aromatic carboxylic acid, BA has multiple health benefits, including antibacterial and anti-inflammatory activities (18). Previously, a research has found that dietary supplementation of BA could increase ADG of weanling pig, but had no obvious difference of feed efficiency (33). In the current study, 1% BA supplementation significantly increased ADG and feed efficiency when compared to 0 BA and 1.5 BA groups. The different reason may be that the animals used in experiment was different. BA can produce esterification products with bacteria, which then affect the metabolism of pathogenic bacteria. Besides, BA can interfere with the DNA activity of pathogenic bacteria, thereby decreasing their growth (34). These effects are conducive to reducing the incidence rate of diarrhea and relieving the negative impact on growth rate of lambs caused by weaning stress. Our results showed that appropriate addition (1%) of BA improved growth performance of weaned lambs. The improved growth performance of animals is usually associated with higher apparent digestibility and nitrogen metabolism. Hence, we conducted the following experiment to study the influence of BA on apparent digestibility and nitrogen metabolism of weaned lambs.

The gastrointestinal tracts of weaned lambs have insufficient digestive enzyme secretion, thus the nutrients digestibility of lambs are low (35). In our study, 0.5 and 1% dietary supplementation of BA significantly increased the nutrients intake of lambs. In general, the typical feeds fed with young ruminants have relatively high values of acid binding capacity, which affect the nutrients intake of animals (36). A previous study found that dietary supplementation with BA could decrease acid binding capacity of feeds (37), which might explain the positive effects of BA on nutrients intake. The digestibility of DM and OM are key parameters to reflect the utilization ability of feed by animals (38). In the current study, the DM and OM digestibility in 1 BA group showed highest values and were significantly elevated as compared with 0 BA group, indicating that the lambs given to 1% BA could obtain more nutrients and accelerate growth, which were matched to ADG results. Organic acid is an effective alternative to enhance nutrient digestibility in animals industry production. The organic acid has multifunctional effects, including reduction of gastrointestinal pH, enhancement of gastrointestinal retention time, stimulation of pancreatic secretions and promotion of gastrointestinal morphology, thus improving nutrient digestibility (39, 40). Our results might relate to the ability of BA to regulate the gastrointestinal pH and digestive enzyme levels and improve gut morphology. In the future, the effects of BA on gastrointestinal development of lambs need in-depth investigation. In addition, we also found that the NDF and ADF digestibility of 1 BA group were higher than 0 and 1.5 BA groups. In ruminants, the microbial community in the rumen is responsible for crude fiber utilization. Unfortunately, the effect of BA on ruminal microbial community has not been reported. A recent study verified that dietary supplementation of BA could stabilize the microbiota fluctuation caused by weaning stress and speed up rapid colonization of dominant bacteria in the gut of piglets (41). Even though the microbial community between monogastric animals and ruminants existed difference, these results indicated that BA could affect the microbial community. We speculated that the positive effects of BA on NDF and ADF digestibility might be associated with the regulation of microbiota in the gastrointestinal tracts. Lastly, the activity of digestive enzyme plays an important role in the nutrients digestion (42). Therefore, more experiments are needed to explore the effects of BA on microbial community and digestive enzyme activity of weaned lambs.

Previously, studies have reported that with the elevation of dietary BA supplementation, the urinary pH was linearly reduced (43), and besides, the HA concentration in urine was linearly increased (44, 45), which also displayed similar findings in our research. The reason for reduced urinary pH may be due to the elevation of urinary HA content. After absorption, the BA is conjugated with glycine through glycine-N-acylase, and then transformed into HA in the liver (46). Moreover, the urea nitrogen concentration in 1% BA supplementation treatments was significantly decreased (3 h), suggesting that the nitrogen conversion of 0 BA lambs was low. As mentioned earlier, the HA synthesis and urea cycle occur in the hepatic mitochondria of ruminants, and together consume NH${\;}_{4}^{+}$ produced from dietary amino acid metabolism (47). Thus, when BA enters the animal's body, does the increase of HA synthesis have the effect of reducing urea production? In the current study, results showed that with the increase of BA intake, the urinary HA content and HA excretion increased linearly, while urinary urea nitrogen content decreased linearly. At the same time, it was found that there was a significant correlation between urinary urea nitrogen excretion and HA nitrogen excretion. This result confirmed our previous hypothesis that the elevation HA excretion by adding BA to the diet reduced urea nitrogen excretion. However, there were unsatisfactory results in terms of total urinary nitrogen output. In addition to hippuric acid nitrogen and urea nitrogen, there are also nitrogen produced by purine derivatives in urinary total nitrogen. In previous studies of crossbred Bulls, it was found that nitrogen produced by purine derivatives (such as allantoin nitrogen) increased linearly as feed intake increased (48). In our experiment, other component nitrogen excretion of weaned lambs increased significantly, and the unsatisfactory total urinary nitrogen excretion might be due to the higher feed intake of 0.5 and 1% BA lambs. In addition, according to our unpublished data, after feeding BA to lactating lambs, metabolites of jejunum contents were mainly enriched in pyrimidine and purine metabolism pathways, indicating that BA intake also affected the metabolism of purine derivatives in lambs. A previous study in rabbits found that intake of sodium benzoate led to an increase in urinary HA excretion and a decrease in urinary urea nitrogen excretion (49), which were consistent with the results of our experiment. Nousiainen et al. (50) reported that increased content of urinary and blood urea nitrogen, indicated a reduced nitrogen efficiency of cows. The 0.5 and 1 BA groups showed higher nitrogen intake when compared to 0 and 1.5 BA groups. According to our results, appropriate supplementation of BA in the diet could improve nitrogen utilization of weaned lambs, which was helpful for promoting growth.

Recently, a study reported that the improvement of ADG was attributed to the elevated nitrogen retention in pigs fed diets with BA (51). In the current study, we did not find obvious difference of nitrogen retention among all groups. However, the 1 BA group had highest value of nitrogen retention. A previous study found that dietary supplementation of BA could improve the intestinal morphology and up-regulate the expression of glucagon-like peptide 2 gene in the intestinal mucous (37), which might enhance nitrogen retention in animals fed ration with BA. We also found that the addition of 1.5% BA to the basal ration decreased nitrogen intake and reduced nitrogen utilization in lambs, thus negatively affecting growth performance. The possible reason might be related to elevation of glycine synthesis required to transform BA into HA in the liver, which might affect the amino acid metabolism and reduce amino acid concentration for synthesis of protein (46). Excessive utilization of protein and amino acid for transformation of HA reduces nitrogen retention. Thus, according to our results, the appropriate supplementation level of BA in the diet of lambs was 1%.

As important indexes related to health condition of animals, the changes of serum biochemistry can be utilized to estimate the body's physiological metabolism and organ functions (52). The T-Bil, D-Bil, ALT, AST, ALP and GT contents in plasma can be used to evaluate the liver function, and the changes of GLU, TG and TC concentrations are associated with fat metabolism. Furthermore, the plasma concentrations of TP and ALB are key parameters of protein metabolism (52). In the present experiment, the plasma contents of GLU, T-Bil, D-Bil, ALT, AST, ALP, GT, TP, TG and TC were similar among four treatments, suggesting that BA supplementation did not have adverse effects on lipid metabolism and hepatic function of weaned lambs. A previous study found that BA supplementation significantly increased the contents of ALB in blood, and reduced the urea nitrogen content (53). Consistent with previous study, our study found that 1% BA supplementation increased plasma ALB content and reduced urea nitrogen concentration, indicating that BA could improve the anabolism of protein to a certain degree, which was conducive to promoting growth of lambs.

Previous studies have verified that the HA is formed by BA biosynthesis reaction and can be excreted from the body's urine within 4 h (54, 55). We studied the dynamic changes of BA, HA and urea nitrogen in plasma of weaned lambs fed BA for the first time. Results showed that after feeding BA 1 h, the plasma concentrations of BA and HA reached up to highest value and then decreased gradually, and the 1 and 1.5 BA groups were higher than other groups. BA is absorbed through the animal's intestine and enters the bloodstream, where it is then transported to the liver for metabolism (13). Our findings indicated that after BA transported to the liver, the process of BA and glycine producing HA under the catalysis of enzyme was very rapid. In animals, BA and glycine are catalyzed by enzymes to form HA, which is then excreted in the urine. In addition, BA can also be metabolized by gut microbes (56, 57). The microbial community of lambs were still in developmental stage, and higher levels of BA and HA may have positive effects on microbial community. However, the potential mechanism of action still needs exploration. As the main products of protein metabolism of animals' body, urea nitrogen are important indexes to reflect protein utilization. The decrease of these contents indicates that the protein utilization is improved, which contributes to nitrogen deposition, and besides, high urea nitrogen content can also have adverse effects on animal health (58). In this study, dietary supplementation of BA effectively decreased plasma urea nitrogen content after morning feed from 2 to 3 h, which was in line with urinary urea nitrogen content mentioned earlier. This result was conducive to improving nitrogen metabolism of lambs. Future research should be paid more attention to the potential mechanisms of BA on the nitrogen metabolism of lambs.

Taken together, BA supplementation had the ability to improve nitrogen metabolism of waned lambs, which was conducive to promoting growth of lambs. However, some limitations need to be acknowledged in the current study. First of all, the absorption pathway of BA in the gastrointestinal tracts requires further elucidation. In the subsequent experiment, we will explore the potential mechanism of action using *in vitro* and *in vivo* studies. In addition, future studies to understand the functions of microbial community in the gastrointestinal tracts using metagenomics or culturomics as well as host function using transcriptomics are needed to provide more information on the role of microorganism in the digestive organ of weaned lambs and their response to dietary BA supplementation.

## 5 Conclusions

Supplementation of BA (1%) improved the ADG, feed efficiency and DM, OM, NDF and ADF digestibility of weaned lambs. In addition, dietary supplementation with 1% BA significantly increased urinary HA contents and excretion as well as nitrogen intake. After supplementation with BA, the plasma and urinary urea nitrogen contents were reduced. Thus, based on our findings, the appropriate supplementation of BA (1%) in the diet improves the growth performance and nitrogen metabolism of weaned lambs.

## Data availability statement

The original contributions presented in the study are included in the article/supplementary material, further inquiries can be directed to the corresponding author.

## Ethics statement

All procedures involving animal care and management used in this experiment were authorized (protocol number: 2020022) by the Institutional Animal Care and Use Committee of Xinjiang Agricultural University (Urumqi, Xinjiang, China). The study was conducted in accordance with the local legislation and institutional requirements.

## Author contributions

WZ: Conceptualization, Investigation, Visualization, Writing – original draft, Writing – review & editing. SS: Investigation, Visualization, Writing – review & editing. YaqZ: Investigation, Writing – review & editing. YanZ: Writing – original draft. JW: Investigation, Writing – review & editing. ZL: Investigation, Writing – review & editing. KY: Conceptualization, Data curation, Funding acquisition, Methodology, Project administration, Supervision, Writing – review & editing.

## References

[1] Worden D Hailu G Jones K Lee YN. The effects of bundling on livestock producers' valuations of environmentally friendly traits available through genomic selection. Can J Agric Econ-Rev Can Agroecon. (2022) 70:263–86. 10.1111/cjag.12322

[2] Cai XH Qin YF Yan BJ Shi WJ. Identification of livestock farms with potential risk of environmental pollution by using a model for returning livestock manure to cultivated land. Environ Sci Pollut Res. (2023) 30:103062–72. 10.1007/s11356-023-29681-5 37676457

[3] Martín-Hernández E Montero-Rueda C Ruiz-Mercado GJ Vaneeckhaute C Martín M. Multi-scale techno-economic assessment of nitrogen recovery systems for livestock operations. Sustain Prod Consump. (2023) 41:49–63. 10.1016/j.spc.2023.07.028 37986715 PMC10659086

[4] van der Weerden TJ Noble AN Beltran I Hutchings NJ Thorman RE de Klein CAM. Influence of key factors on ammonia and nitrous oxide emission factors for excreta deposited by livestock and land-applied manure. Sci Total Environ. (2023) 889:164066. 10.1016/j.scitotenv.2023.164066 37201844

[5] Soares JR Souza BR Mazzetto AM Galdos MV Chadwick DR Campbell EE. Mitigation of nitrous oxide emissions in grazing systems through nitrification inhibitors: a meta-analysis. Nutr Cycl Agroecosyst. (2023) 19:10256–8. 10.1007/s10705-022-10256-8

[6] Cheng LX Zhang XM Reis S Ren CC Xu JM Gu BJ. 12% switch from monogastric to ruminant livestock production can reduce emissions and boost crop production for 525 million people. Nat Food. (2023) 4:190–210. 10.1038/s43016-022-00661-1 37118312

[7] Jin D Zhao SG Zheng N Beckers Y Wang JQ. Urea metabolism and regulation by rumen bacterial urease in ruminants-a review. Ann Anim Sci. (2018) 18:303–18. 10.1515/aoas-2017-0028

[8] Ewaoluwagbemiga EO Bee G Kasper C. Genetic analysis of protein efficiency and its association with performance and meat quality traits under a protein-restricted diet. Genet Sel Evol. (2023) 55:16. 10.1186/s12711-023-00812-3 37268880 PMC10236592

[9] Seleem MS Wu ZH Xing CQ Zhang Y Hanigan MD Bu DP. Impacts of rumen-encapsulated methionine and lysine supplementation and low dietary protein on nitrogen efficiency and lactation performance of dairy cows. J. Dairy Sci. (2023). 10.3168/jds.2023-23404 37923213

[10] Hristov AN Bannink A Crompton LA Huhtanen P Kreuzer M McGee M. Invited review: nitrogen in ruminant nutrition: a review of measurement techniques. J. Dairy Sci. (2019) 102:5811–52. 10.3168/jds.2018-15829 31030912

[11] McCoard SA Pacheco D. The significance of N-carbamoylglutamate in ruminant production. J Anim Sci Biotechnol. (2023) 14:17. 10.1186/s40104-023-00854-z 37046347 PMC10100185

[12] Ticinesi A Guerra A Nouvenne A Meschi T Maggi S. Disentangling the complexity of nutrition, frailty and gut microbial pathways during aging: a focus on hippuric acid. Nutrients. (2023) 15:05118. 10.3390/nu15051138 36904138 PMC10005077

[13] Ulaszewska M Garcia-Aloy M Vázquez-Manjarrez N Soria-Florido MT Llorach R Mattivi F. Food intake biomarkers for berries and grapes. Genes Nutr. (2020) 15:35. 10.1186/s12263-020-00675-z 32967625 PMC7509942

[14] De Simone G Balducci C Forloni G Pastorelli R Brunelli L. Hippuric acid: could became a barometer for frailty and geriatric syndromes? Ageing Res Rev. (2021) 72:101466. 10.1016/j.arr.2021.101466 34560280

[15] Doak BW. Some chemical changes in the nitrogenous constituents of urine when voided on pasture. J Agr Sci. (1952) 42:162–71. 10.1017/S0021859600058767

[16] Bristow AW Whitehead DC Cockburn JE. Nitrogenous constituents in the urine of cattle, sheep and goats. J Sci Food Agr. (1992) 59:387–94. 10.1002/jsfa.2740590316

[17] Rychen G Aquilina G Azimonti G Bampidis V de Lourdes Bastos M Bories G. Safety and efficacy of VevoVitall^®^(benzoic acid) as feed additive for minor porcine species. EFSA J. (2017) 15:e05026. 10.2903/j.efsa.2017.5026 32625315 PMC7009800

[18] Süloglu AK Koçkaya EA Selmanoglu G. Toxicity of benzyl benzoate as a food additive and pharmaceutical agent. Toxicol Ind Health. (2022) 38:221–33. 10.1177/07482337221086133 35332820

[19] Gao Z Yu B Zheng P He J Mao X Yu J. Effects of Benzoic acid on intestinal microflora and metabolites of piglets. Chin J Anim Nutr. (2014) 26:1044–54.

[20] Chen J Chen D Yu B He J Mao X Yu J. Effects of Benzioc acid on growth performance, organ indexes and gastrointestinal content pH of weaned piglets. Chin J Anim Nutr. (2015) 27:238–46.

[21] Mao XB Yang Q Chen DW Yu B He J. Benzoic acid used as food and feed additives can regulate gut functions. Biomed Res Int. (2019) 2019:6. 10.1155/2019/5721585 30931328 PMC6413358

[22] Diao H Zheng P Yu B He J Mao XB Yu J Chen DW. Effects of dietary supplementation with benzoic acid on intestinal morphological structure and microflora in weaned piglets. Livest Sci. (2014) 167:249–56. 10.1016/j.livsci.2014.05.029

[23] Hailemariam S Zhao SG He Y Wang JQ. Urea transport and hydrolysis in the rumen: a review. Anim Nutr. (2021) 7:989–96. 10.1016/j.aninu.2021.07.002 34738029 PMC8529027

[24] Council NR. Nutrient Requirements of Small Ruminants. Washington, DC: The National Academies Press (2007).

[25] Williams MS Mandell IB Bohrer BM Wood KM. The effects of feeding benzoic acid and/or live active yeast (Saccharomyces cerevisiae) on beef cattle performance, feeding behavior, and carcass characteristics. Transl Anim Sci. (2021) 5:txab143. 10.1093/tas/txab143 34877478 PMC8643465

[26] O'Meara FM Gardiner GE O'Doherty JV Lawlor PG. Effect of dietary inclusion of benzoic acid (VevoVitall^®^) on the microbial quality of liquid feed and the growth and carcass quality of grow-finisher pigs. Livest Sci. (2020) 237:104043. 10.1016/j.livsci.2020.104043

[27] López MC Fernández C. Energy partitioning and substrate oxidation by Murciano-Granadina goats during mid lactation fed soy hulls and corn gluten feed blend as a replacement for corn grain. J Dairy Sci. (2013) 96:4542–52. 10.3168/jds.2012-6473 23628256

[28] AOAC. Association of Offical Analytical Chemists Official Methods of Analysis. Arlington, VA (2010).

[29] Aguiar FD Bezerra LR Cordao MA Cavalcante ITR de Oliveira JPF do Nascimento RR. Effects of increasing levels of total tannins on intake, digestibility, and balance of nitrogen, water, and energy in hair lambs. Animals. (2023) 13:2497. 10.3390/ani13152497 37570305 PMC10416999

[30] Kubota K Horai Y Kushida K Ishizaki T. Determination of benzoic acid and hippuric acid in human plasma and urine by high-performance liquid chromatography. J Chromatogra B. (1988) 425:67–75. 10.1016/0378-4347(88)80007-0 3360879

[31] Guo YX Yang RC Duan CH Wang Y Hao QH Ji SK. Effect of dioscorea opposite waste on growth performance, blood parameters, rumen fermentation and rumen bacterial community in weaned lambs. J Integr Agric. (2023) 22:1833–46. 10.1016/j.jia.2022.10.002

[32] Mao HL Ji WW Yun Y Zhang YF Li ZF Wang C. Influence of probiotic supplementation on the growth performance, plasma variables, and ruminal bacterial community of growth-retarded lamb. Front Microbiol. (2023) 14:1216534. 10.3389/fmicb.2023.1216534 37577421 PMC10413120

[33] Warner AJ DeRouchey JM Tokach MD Woodworth JC Goodband RD Gebhardt JT. Effect of added calcium carbonate without and with benzoic acid on weanling pig growth performance, fecal dry matter, and blood Ca and P concentrations. Transl Anim Sci. (2023) 7:txad055. 10.1093/tas/txad055 37415595 PMC10319757

[34] Rohatgi A Gupta P. Benzoic acid derivatives as potent antibiofilm agents against Klebsiella pneumoniae biofilm. J Biosci Bioeng. (2023) 136:190–7. 10.1016/j.jbiosc.2023.06.011 37479559

[35] Abdelsattar MM Vargas-Bello-Perez E Zhuang YM Fu YZ Zhang NF. Impact of dietary supplementation of β-hydroxybutyric acid on performance, nutrient digestibility, organ development and serum stress indicators in early-weaned goat kids. Anim Nutr. (2022) 9:16–22. 10.1016/j.aninu.2021.11.003 35949983 PMC9344317

[36] Wang Y Lu N Tian X Yang J Chen Y Bai C. Effects of acid-binding capacity levels in diet on performance and nutrient apparent digestibility of lambs. Chin J Anim Nutr. (2009) 21:488–92.

[37] Diao H Gao Z Yu B Zheng P He J Yu J. Effects of benzoic acid (Vevo Vitall) on the performance and jejunal digestive physiology in young pigs. J Anim Sci Biotechnol. (2016) 7:32. 10.1186/s40104-016-0091-y 27239300 PMC4884408

[38] Ma J Fan X Zhang W Zhou G Yin F Zhao Z. Grape seed extract as a feed additive improves the growth performance, ruminal fermentation and immunity of weaned beef calves. Animals. (2023) 13:1876. 10.3390/ani13111876 37889835 PMC10251878

[39] Han MM Chen BB Dong YY Miao ZQ Su Y Liu C. Evaluation of liquid organic acids on the performance, Chyme pH, nutrient utilization, and gut microbiota in broilers under high stocking density. Animals. (2023) 13:17. 10.3390/ani13020257 36670796 PMC9854823

[40] Li ZQ Liu SH Zhao YR Wang JY Ma XK. Compound organic acid could improve the growth performance, immunity and antioxidant properties, and intestinal health by altering the microbiota profile of weaned piglets. J Anim Sci. (2023) 101:12. 10.1093/jas/skad196 37314321 PMC10355368

[41] Wei K Yang X Zhao H Chen H Bei W. Effects of combined application of benzoic acid and 1-monolaurin on growth performance, nutrient digestibility, gut microbiome and inflammatory factor levels in weaned piglets. Porc Health Manag. (2023) 9:46. 10.1186/s40813-023-00339-5 37858213 PMC10588023

[42] Ma J Zhu X Wang Z Yu X Hu R Wang X. Glutamine supplementation affected the gut bacterial community and fermentation leading to improved nutrient digestibility in growth-retarded yaks. FEMS Microbiol. Ecol. (2021) 97:fiab084. 10.1093/femsec/fiab084 34132351

[43] Halas D Hansen CF Hampson DJ Mullan BP Kim JC Wilson RH. Dietary supplementation with benzoic acid improves apparent ileal digestibility of total nitrogen and increases villous height and caecal microbial diversity in weaner pigs. Anim Feed Sci Technol. (2011) 163:261–7. 10.1016/j.anifeedsci.2010.11.012

[44] Gräber T Kluge H Hirche F Broz J Stangl GI. Effects of dietary benzoic acid and sodium-benzoate on performance, nitrogen and mineral balance and hippuric acid excretion of piglets. Arch Anim Nutr. (2012) 66:227–36. 10.1080/1745039X.2012.676812 22724168

[45] Kluge H Broz J Eder K. Effects of dietary benzoic acid on urinary pH and nutrient digestibility in lactating sows. Livest Sci. (2010) 134:119–21. 10.1016/j.livsci.2010.06.116

[46] Bühler K Wenk C Broz J Gebert S. Influence of benzoic acid and dietary protein level on performance, nitrogen metabolism and urinary pH in growing-finishing pigs. Arch Anim Nutr. (2006) 60:382–9. 10.1080/17450390600884369 17036747

[47] Schuba J Südekum KH Pfeffer E Jayanegara A. Excretion of faecal, urinary urea and urinary non-urea nitrogen by four ruminant species as influenced by dietary nitrogen intake: a meta-analysis. Livest Sci. (2017) 198:82–8. 10.1016/j.livsci.2017.01.017

[48] George SK Dipu MT Mehra UR Verma AK Singh P. Influence of level of feed intake on concentration of purine derivatives in urinary spot samples and microbial nitrogen supply in crossbred bulls. Asian Australas J Anim Sci. (2006) 19:1291–7. 10.5713/ajas.2006.1291

[49] Lewis HB. Studies on the synthesis of hippuric acid in the animal organism. I the synthesis of hippuric acid in rabbits on a glycocoll-free diet. J Biol Chem. (1914) 17:503–8. 10.1016/S0021-9258(18)88391-0

[50] Nousiainen J Shingfield KJ Huhtanen P. Evaluation of milk urea nitrogen as a diagnostic of protein feeding. J Dairy Sci. (2004) 87:386–98. 10.3168/jds.S0022-0302(04)73178-1 14762082

[51] Choi H Chen Y Longo F Kim SW. Comparative effects of benzoic acid and sodium benzoate in diets for nursery pigs on growth performance and acidification of digesta and urine. J Anim Sci. (2023) 101:skad116. 10.1093/jas/skad116 37115097 PMC10184693

[52] Huang YH Yan Q Jiang MC Guo S Li HW Lin M. Astragalus membranaceus additive improves serum biochemical parameters and reproductive performance in postpartum dairy cows. Front Vet Sci. (2022) 9:952137. 10.3389/fvets.2022.952137 35898551 PMC9310658

[53] Shu Y Yu B He J Yu J Zheng P Yuan ZC. Excess of dietary benzoic acid supplementation leads to growth retardation, hematological abnormality and organ injury of piglets. Livest Sci. (2016) 190:94–103. 10.1016/j.livsci.2016.06.010

[54] Shin MY Shin C Choi JW Lee J Lee S Kim S. Pharmacokinetic profile of propyl paraben in humans after oral administration. Environ Int. (2019) 130:9. 10.1016/j.envint.2019.104917 31234001

[55] Baba S Akira K Suzuki H Imachi M. Use of nuclear magnetic resonance spectroscopy and selective C-labeling for pharmacokinetic research in man: detection of benzoic acid conversion to hippuric acid. Biol Pharm Bull. (1995) 18:643–7. 10.1248/bpb.18.643 7492975

[56] Wang Y Chiba LI Huang C Torres IM Wang L Welles EG. Effect of diet complexity, multi-enzyme complexes, essential oils, and benzoic acid on weanling pigs. Livest Sci. (2018) 209:32–8. 10.1016/j.livsci.2017.12.007

[57] Han X Li MM Sun LJ Liu XJ Yin Y Hao JY. p-Hydroxybenzoic acid ameliorates colitis by improving the mucosal barrier in a gut microbiota-dependent manner. Nutrients. (2022) 14:5383. 10.3390/nu14245383 36558542 PMC9784546

[58] Recavarren MI Milano GD. The rate and pattern of urea infusion into the rumen of wethers alters nitrogen balance and plasma ammonia. J Anim Physiol Anim Nutr. (2014) 98:1047–53. 10.1111/jpn.12168 24611997

